# Annexin A1 Contained in Extracellular Vesicles Promotes the Activation of Keratinocytes by Mesoglycan Effects: An Autocrine Loop Through FPRs

**DOI:** 10.3390/cells8070753

**Published:** 2019-07-19

**Authors:** Emanuela Pessolano, Raffaella Belvedere, Valentina Bizzarro, Paola Franco, Iolanda De Marco, Francesco Petrella, Amalia Porta, Alessandra Tosco, Luca Parente, Mauro Perretti, Antonello Petrella

**Affiliations:** 1 Department of Pharmacy, University of Salerno, via Giovanni Paolo II 132, 84084 Fisciano, Italy; 2 Department of Industrial Engineering, University of Salerno, via Giovanni Paolo II 132, 84084 Fisciano, Italy; 3 Primary Care-Wound Care Service, Health Local Agency Naples 3 South, Via Libertà 42, 80055 Portici, Italy; 4 The William Harvey Research Institute, Barts and The London School of Medicine and Dentistry, Queen Mary University of London, London EC1M 6BQ, UK

**Keywords:** annexin A1, EVs, keratinocytes, mesoglycan, skin wound healing

## Abstract

We have recently demonstrated that mesoglycan, a fibrinolytic compound, may be a promising pro-healing drug for skin wound repair. We showed that mesoglycan induces migration, invasion, early differentiation, and translocation to the membrane of keratinocytes, as well as the secretion of annexin A1 (ANXA1), further involved in keratinocytes activation. These events are triggered by the syndecan-4 (SDC4)/PKCα pathway. SDC4 also participates to the formation and secretion of microvesicles (EVs) which may contribute to wound healing. EVs were isolated from HaCaT cells, as human immortalized keratinocytes, and then characterised by Western blotting, Field Emission-Scanning Electron Microscopy, and Dynamic Light Scattering. Their autocrine effects were investigated by Wound-Healing/invasion assays and confocal microscopy to analyse cell motility and differentiation, respectively. Here, we found that the mesoglycan increased the release of EVs which amplify its same effects. ANXA1 contained in the microvesicles is able to promote keratinocytes motility and differentiation by acting on Formyl Peptide Receptors (FPRs). Thus, the extracellular form of ANXA1 may be considered as a link to intensify the effects of mesoglycan. In this study, for the first time, we have identified an interesting autocrine loop ANXA1/EVs/FPRs in human keratinocytes, induced by mesoglycan.

## 1. Introduction

Skin injury triggers a cascade of molecular and cellular events directed to the restoration of the physiological barrier. These events take place in three overlapping phases, namely inflammatory, proliferative, and remodelling phases. The proliferative moment is characterized by re-epithelialization, during which the main actors are keratinocytes. The activation of these cells is followed by proliferation and migration at the wounded edges, next, the epithelial regeneration finishes when keratinocytes gradually differentiate to form the stratum corneum [1,2].

Intense research efforts are currently directed to the development of new drugs and technologies to promote re-epithelialization in tissue repair. Our studies focused on the use of mesoglycan as a novel therapeutic strategy. Mesoglycan is a mixture of glycosaminoglycans (GAGs) extracted from the porcine intestinal mucosa and includes heparan sulfate (HS), dermatan sulfate (DS), slow-moving heparin (HEP), and chondroitin sulfate (CS). It is mainly known as antithrombotic and pro-fibrinolytic compound [3,4], but our previous works showed its beneficial effects on the re-epithelialization phase [5,6]. A characterized biochemical mechanism by which mesoglycan influences keratinocytes behaviour is carried out through the activation of the syndecan 4 (SDC4), the fourth characterized member of the family of heparan sulfate proteoglycans (HSPGs), that acts as a trans-membrane coreceptor [7]. Particularly, our recent study showed that both migration/invasion and differentiation were negatively affected by SDC4 loss of function on keratinocytes even when treated with mesoglycan. Moreover, dependent on SDC4 activation, annexin A1 (ANXA1) forms a complex with S100A11 at the inner surface of the plasma membrane [8].

ANXA1 is the first characterized member of the annexins family, Ca^2+^-dependent phospholipid-binding proteins involved in numerous physiopathological processes [9]. Among its functions, ANXA1 is also known as one of the mediators of cell motility because it participates in membrane–cytoskeleton organization [10]. Furthermore, ANXA1 is important in keratinocytes differentiation, during which the protein participates in the cornified envelope formation. In this event ANXA1 is found to be a substrate for transglutaminase which catalyzes the crosslinking of soluble cytoplasmic substrates, known as cornified envelope precursors [11]. The sub-cellular localization of ANXA1 is a crucial issue for its role [12]. In our system, following the administration of mesoglycan to human keratinocytes, ANXA1 translocates to the plasma membrane and then it is released to the extracellular environment [8]. Once secreted, the protein acts in an autocrine and paracrine manner through Formyl-Peptide Receptors (FPRs), and it is able to exert therapeutic functions on wound closure [13,14].

ANXA1 lacks specific sequences able to cross the phospholipid membrane, so the mechanism by which it is secreted is still studied. ANXA1 could be externalized through direct interaction with plasma membrane or membrane transporters. Actually, the protein plays an important intracellular role in vesicle-trafficking, mediating the interaction of vesicles with cytoplasmic machinery, for the production of extracellular vesicles (EVs), particularly exosomes [15].

EVs, such as exosomes, released by different cell types, including keratinocytes, contribute to beneficial actions in several physiopathological processes, even in tissue repair. However, the exact mechanisms by which these vesicles could promote the reconstitution of skin barrier is still unclear [16]. Furthermore, SDC receptors are involved in the biogenesis of exosomes [17].

In this work, we demonstrate that human keratinocytes, when treated with mesoglycan, secrete a huge amount of EVs containing the pro-resolving mediator ANXA1. Next, using the ANXA1 mimetic peptide Ac2-26 we confirm that the extracellular form of this protein is able to promote keratinocytes motility and differentiation creating an autocrine loop through FPRs.

## 2. Materials and Methods

### 2.1. Cell Cultures

HaCaT cell line (human immortalized keratinocytes spontaneously established from a transformed human epithelial cell line of adult skin) was purchased from CLS Cell Lines Service GmbH (Eppelheim, Germany) and was maintained in Dulbecco’s modified Eagle’s medium (DMEM, Euroclone, Milan, Italy) with 10% fetal bovine serum (FBS, Euroclone, Milan, Italy) following the instructions reported in [18]. The medium was supplemented with antibiotics (10,000 U/mL penicillin and 10 mg/mL streptomycin, Euroclone, Milan, Italy); cells were maintained at 37 °C in 5% CO_2_–95% air humidified atmosphere and were serially passed at 70–80% confluence.

### 2.2. Exosome Enrichment

The enrichment of exosomes from cell culture supernatants has been performed as reported in [19]. HaCaT cells (1.5 × 10^5^ cm^−2^, for a total of about 8 × 10^7^ cells) were incubated for 24 h in DMEM medium without FBS treated or not with mesoglycan (0.3 mg/mL, kindly provided by LDO, *Laboratori Derivati Organici spa*, Vercelli, Italy). Conditioned medium (also reported as SS) was collected and centrifuged for 5 min at 300× *g* at room temperature (RT) to remove detached cells; the supernatant was transferred and centrifuged for 10 min at 2000× *g* at 4 °C to remove dead cells. The obtained supernatant was transferred and centrifuged at 10,000× *g* for 30 min at 4 °C to eliminate cell debris. Then, the cleared supernatant was transferred to ultracentrifuge tubes and centrifuged for 70 min at 100,000× *g* at 4 °C. Next, the supernatant was stored and used as EDS (EVs-depleted supernatant); the pellet was washed in PBS and re-ultracentrifuged at 100,000× *g* at 4 °C for 70 min. Finally, the supernatant was removed and the pellet was resuspended. The buffer we selected for the resuspension was sterile bidistilled water with 5 mM EDTA, to avoid vesicles aggregation, for FE-SEM (Field Emission-Scanning Electron Microscope) and DLS (dynamic light scattering) analysis, 50 µL RIPA lysis buffer for Western blotting, or 200 µL PBS for the administration to cells. The normalization through Bradford assay has been performed using the correspondent amount of EVs lysed in RIPA buffer. This normalization has been important for us in order to administrate to cells the same amount of EVs (20 µg of proteins), derived from HaCaT treated with mesoglycan or not (EVs mesoglycan and EVs ctrl, respectively), on all the experimental points. All analyses were performed on fresh isolated fractions.

### 2.3. Field Emission-Scanning Electron Microscope (FE-SEM) Analysis

Sample morphology was analysed using a FE-SEM model LEO 1525 (Carl Zeiss SMTAG; Oberkochen, Germany). The EVs enriched in exosomes were fixed with 2% *v/v* p-formaldehyde and 1% *v/v* glutaraldehyde (Sigma-Aldrich; Saint Louis, MO, USA) in PBS. Next, a drop of the suspension was spread on a carbon tab placed on an aluminium stub (Agar Scientific; Stansted, UK) and left to dry in a stream of nitrogen for 25 min. Then, the dried samples were coated with gold (layer thickness 250 Å) using a sputter coater (model 108 A, Agar Scientific; Stansted, UK). Each analysis was performed in triplicate.

### 2.4. Dynamic Light Scattering (DLS) Analysis

The DLS technique was performed using a Zetasizer Nano S instrument (Worcestershire, UK) in order to obtain particle size distribution by number of the EVs. The DLS instrument works at 25 °C and is equipped with a 5.0 mW He-Ne laser operating at 633 nm with a scattering angle of 173°. Each measurement was repeated in triplicate.

### 2.5. Western Blotting

Protein expression was examined by SDS-PAGE, as described previously [20]. Briefly, total intracellular proteins were extracted from the cells by freeze/thawing in lysis buffer containing protease inhibitors. Protein content was estimated according to Biorad protein assay (BIO-RAD). A total of 20 µg of proteins were visualized using the chemioluminescence detection system (Amersham biosciences; Little Chalfont, UK) after incubation with rabbit polyclonal primary antibodies against ANXA1 (1:10,000; Invitrogen; Carlsbad, CA, USA) and calreticulin (1:1000; Elabscience; Houston, TX, USA), with mouse monoclonal primary antibodies against TSG101 (1:1000; ThermoFisher Scientific; Waltham, MA, USA) and anti-β actin (mouse monoclonal; 1:1000; clone AC15; A5441, Sigma-Aldrich). The blots were exposed and analysed to Las4000 (GE Healthcare Life Sciences).

### 2.6. Invasion Assay

Cell invasiveness was studied using the Trans-well Cell Culture (12 mm diameter, 8.0-fim pore size) purchased from Corning Incorporated (New York, NY, USA), as previously described [20]. Briefly, 7 × 10^4^ HaCaT cells were plated in 350 µL of medium serum-free in the upper chamber of the trans-well. 1,4 mL of DMEM with FBS and with or without Ac2-26 and Boc-1 were put in the lower chamber and the trans-well was left for 24 h at 37 °C in 5% CO_2_–95% air humidified atmosphere. After 24 h, the Trans-well Cell Culture chambers were washed twice with PBS and fixed with 4% p-formaldehyde for 10 min, and then with 100% methanol for 20 min. Later, the fixed cells were stained with crystal violet (0.5% *w/v* in a *v/v* solution of 20% methanol/distilled water; Merck Chemicals, Darmstadt, Germany) for 15 min. Next, the chambers were washed again in PBS and cleaned with a cotton bud to remove crystal violet exceedance. All of the experimental points were treated with mitomycin C (10 μg/mL, Sigma-Aldrich, St. Louis, MO, USA) to ensure the block of mitosis. The number of cells that had migrated to the lower surface was counted in twelve random fields using EVOS light microscope (10×) (Life technologies Corporation, Carlsbad, CA, USA).

### 2.7. Confocal Microscopy

HaCaT cells, fixed in p-formaldehyde (4% *v/v* in PBS; Lonza; Basilea, Switzerland), were permeabilized with Triton X-100 (0.5% *v/v* in PBS; Lonza; Basilea, Switzerland), blocked with goat serum (20% *v/v* in PBS; Lonza; Basilea, Switzerland), and then incubated with anti-ANXA1 antibody (rabbit polyclonal; 1:100; Invitrogen; Carlsbad, CA, USA), anti-CK6 (rabbit polyclonal; 1:500; Flarebio Biotech LLC., MD, USA), anti-CK10 (rabbit polyclonal; 1:500; Flarebio Biotech LLC., MD, USA), anti-involucrin (mouse monoclonal; 1:250; Santa Cruz Biotechnologies, CA, USA), anti-E-cadherin (mouse monoclonal; 1:500; BD Biosciences, Franklin Lakes, NJ, USA) O/N at 4 °C. The staining with the AlexaFluor 488/550 anti-rabbit and anti-mouse antibodies (1:1000; Molecular Probes, Eugene, OR, USA) and for the nuclei and the following confocal analysis were performed as reported in [21]. Fluorescence intensity analyses were performed using ImageJ software (NIH, Bethesda, MD, USA) as following described. Briefly, ten field images from a single coverslip were randomly selected for three coverslips and registered for each experimental condition identifying distinct cells by Hoechst 33342 nuclear staining. Then, individual cell total area was selected using an area selection tool and fluorescence intensity value was measured subtracting background. The obtained mean value was used to compare experimental groups. Quantifications were performed from multichannel images obtained using a 63× objective using ImageJ, marking either the cell perimeter or the nucleus as the region of interest and calculating integrated densities per area from the appropriate channel. A minimum of 50 cells were analysed for each data set. The obtained mean value was used to compare experimental groups.

### 2.8. Supernatant, Cytosol and Membrane Extracts

Compartimentalized protein extracts were obtained as reported [22]. Briefly, HaCaT growth media were harvested, frozen at −80 °C, and lyophilized. Dried samples were suspended in lysis buffer containing protease inhibitors and left at 4 °C for 30 min. After centrifugation, the supernatants represented the protein sample. Additionally, HaCaT cells were washed twice with PBS, detached with trypsin-EDTA 1× in PBS, harvested in PBS and centrifuged for 5 min at 600× *g* at 4 °C. After that, cells were lysed in 4 mL of buffer A (Tris HCl 20 mM, pH 7, 4; sucrose 250 mM; DTT 1 mM; protease inhibitors, EDTA 1 mM in water), sonicated (5 s pulse–9 s pause for 2 min, amplitude 42%), and then centrifuged at 4 °C for 10 min, at 5000 × *g*. The resulting supernatants were ultra-centrifuged for 1 h at 100,000× *g* at 4 °C, until new supernatants were obtained corresponding to cytosol extracts. Each resultant pellet was dissolved in 4 mL of buffer A and ultra-centrifuged for 1 h at 100,000× *g* at 4 °C. The pellets were then resuspended in 250 μL of buffer B (Tris HCl 20 mM, pH 7, 4; DTT 1 mM; EDTA 1 mM; Triton X-100 1%, in water) and left overnight on orbital shaker at 4 °C. Next, the solution was centrifuged for 30 min at 50,000× *g* at 4 °C: the supernatants represent membrane extracts.

### 2.9. In Vitro Wound-Healing Assay

HaCaT cells were seeded in a 12-well plastic plate at 5 × 10^5^ cells for well. After 24 h incubation, cells reached 100% confluency and a wound was produced at the center of the monolayer by gently scraping the cells with a sterile plastic p10 pipette tip to create a wound area of about 500 µm. After removing incubation medium and washing with PBS, cell cultures were incubated in the presence of EVs ctrl, EVs mesoglycan, Ac2-26 (1 μM; Tocris Bioscience, Bristol, UK), Boc-1 (100 μM; Bachem AG, Bubendorf, Switzerland), or in growth medium as control. All experimental points were further treated with mitomycin C (10 μg/mL, Sigma Aldrich, St. Louis, MO, USA) to ensure the block of mitosis. The wounded cells were then incubated at 37 °C in a humidified and equilibrated (5% *v/v* CO_2_) incubation chamber of an Integrated Live Cell Workstation Leica AF-6000 LX (Leica Microsystems, Wetzlar, Germany). A 10× phase contrast objective was used to record cell movements with a frequency of acquisition of 10 min on at least 10 different positions for each experimental condition. The migration rate of individual cells was determined by measuring the wound closure from the initial time to the selected time-points (bar of distance tool, Leica ASF software, version Lite 2.3.5, Leica microsystem CMS Gmvh). For each wound 5 different positions were registered, and for each position 10 different cells were randomly selected to measure the migration distances.

### 2.10. Measurement of Intracellular Ca^2+^ Signalling

Intracellular Ca^2+^ concentrations [Ca^2+^] were measured using the fluorescent indicator dye Fura 2-AM (Sigma Aldrich, St. Louis, MO, USA), the membrane-permeant acetoxymethyl ester form of Fura 2, as previously described [23]. Briefly, HaCaT cells (1 × 10^5^/mL) were washed in (PBS) resuspended in 1 mL of Hank’s balanced salt solution (HBSS, Thermo Fisher Scientific, Waltham, MA, USA) containing 5 µM Fura 2-AM and incubated for 45 min at 37 °C. After the incubation period, cells were washed with the same buffer to remove excess of Fura 2-AM and then incubated in 1 mL of buffer. Keratinocytes were then transferred to the spectrofluorimeter (Perkin-Elmer LS-55, Waltham, MA, USA). Treatments with ionomycin (1 mM; Sigma Aldrich, St. Louis, MO, USA), EDTA (15 mM, Sigma Aldrich, St. Louis, MO, USA), fMLP (50 nM; Sigma Aldrich, St. Louis, MO, USA), Ac2-26 (1 µM), Boc-1 (100 µM) were carried out by adding the appropriate concentrations of each substance into the cuvette in Ca^2+^-free HBSS/0.5 mM EDTA buffer. The excitation wavelength was alternated between 340 and 380 nm, and emission fluorescence was recorded at 515 nm. The fluorescence ratio was calculated as F340/F380 nm. Maximum and minimum [Ca^2+^] were determined at the end of each experimental protocol by adding to the cells HBSS containing 1 mM ionomycin and 15 mM EDTA, respectively, according to the equation of Grynkiewicz [24].

### 2.11. Statistical Analysis

Data analyses and statistical evaluations were carried out using Microsoft Excel; the number of independent experiments and p-values are indicated in the figure legends. All results are the mean ± standard deviation of at least 3 experiments performed in triplicate. Statistical comparisons between the experimental points were made using two-tailed *t*-test comparing two variables. Differences were considered significant if *p* < 0.05, *p* < 0.01 and *p* < 0.001.

## 3. Results

### 3.1. Mesoglycan Promoted the Release of Extracellular Vesicles from Keratinocytes

The paracrine activity of EVs has raised great interest in wound repair. Particularly, the microvesicles-mediated interactions play key roles in the progression of the normal skin wound healing sequence [25].

**Figure 1:**
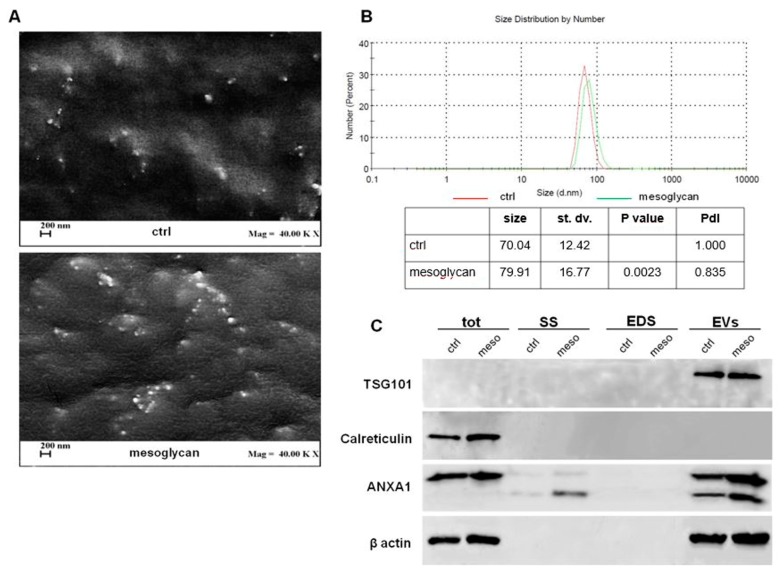
Evaluation of extracellular vesicles (EVs) isolated from keratinocytes. (**A**) EVs deriving from keratinocytes treated or not with mesoglycan were imaged by Field Emission-Scanning Electron Microscope (FE-SEM). Magnitude = 40,000 KX (that is 40,000,000) and scale bar = 200 nm. (**B**) Characterization of the EVs size distributions was found by dynamic light scattering (DLS). The table reports the EVs mean size, standard deviation, *p* value, referring to EVs mesoglycan distribution vs. EVs ctrl, and PdI. The experiments were performed in triplicate. (**C**) Western blot using antibodies against TSG101, calreticulin, and ANXA1 on protein content of total cell lysates, conditioned medium (SS), EV-depleted extracellular fractions (EDS) and EVs fractions extracted from keratinocytes treated or not with mesoglycan. Protein normalization and the check of the sample quality were performed on β-actin levels.

In this study, we used mesoglycan on HaCaT cells as reported in [5], in order to investigate its role in EVs secretion. Thus, we purified EVs, enriched in exosomes, by supernatant of cells and analysed them by Field Emission Scanning Electron Microscopy (FE-SEM) and dynamic light-scattering (DLS). In the first case we showed rounded particles ranging from 30 to 180 nm in diameter which present the typical morphological features of exosomes (Figure 1A). Furthermore, we found that keratinocytes secreted an increased amount of EVs when treated with mesoglycan. This result has been corroborated through DLS, by which we estimated the EVs size distribution by number depending on the measurement of the diameter. In Figure 1B, the red curve refers to EVs released by control HaCaT, the green one to the same cells treated with mesoglycan. The table reports the size means, the standard deviation (st. dv.), the *p* value, refers to the distribution of EVs secreted by keratinocytes treated with mesoglycan (EVs mesoglycan) versus control ones (EVs ctrl), and the Polydispersity Index (PdI). In the area under the curves are comprised 99.3% and 99.0% of the values derived from the analysis of EVs ctrl and EVs mesoglycan samples, respectively. Among these values, ranging from about 30 to 180 nm, the mean diameter is 90 nm. 

In order to confirm the enrichment in exosomes of the EVs isolated from HaCaT cells, Western blot analysis was conducted as reported in [15]. Figure 1C shows the presence of TSG101 exclusively in EV fractions and of calreticulin only in total cell lysates. Indeed, TSG101 is frequently used as an exosome marker since it is involved in their biogenesis, maturation, and secretion, while calreticulin is exposed on the surface of apoptotic cells and ends up in apoptotic bodies. Finally, the presence of ANXA1 was detected in total cell lysate, in conditioned medium (SS) and in EVs. Specifically, in SS and EVs, ANXA1 appeared as full length protein (37 kDa) and in its cleaved form (33 kDa). Moreover, SS and EVs mesoglycan showed a larger amount of ANXA1 if compared to SS and EVs ctrl.

### 3.2. EVs Isolated from Keratinocytes Treated with Mesoglycan Increased the Invasive Ability and the Differentiation of the Same Keratinocytes

The interaction of microvesicles with target cells triggers intracellular events that control a myriad of cellular responses, such as proliferation, survival, migration, adhesion, and differentiation [25]. However, the functions in wound repair of EVs deriving from skin cells are still not clear. In order to evaluate the autocrine effects of microvesicles isolated by HaCaT cells treated or not with mesoglycan (EVs mesoglycan and EVs ctrl, respectively), an invasion assay by administering this EVs on the same keratinocytes was performed. 

**Figure 2:**
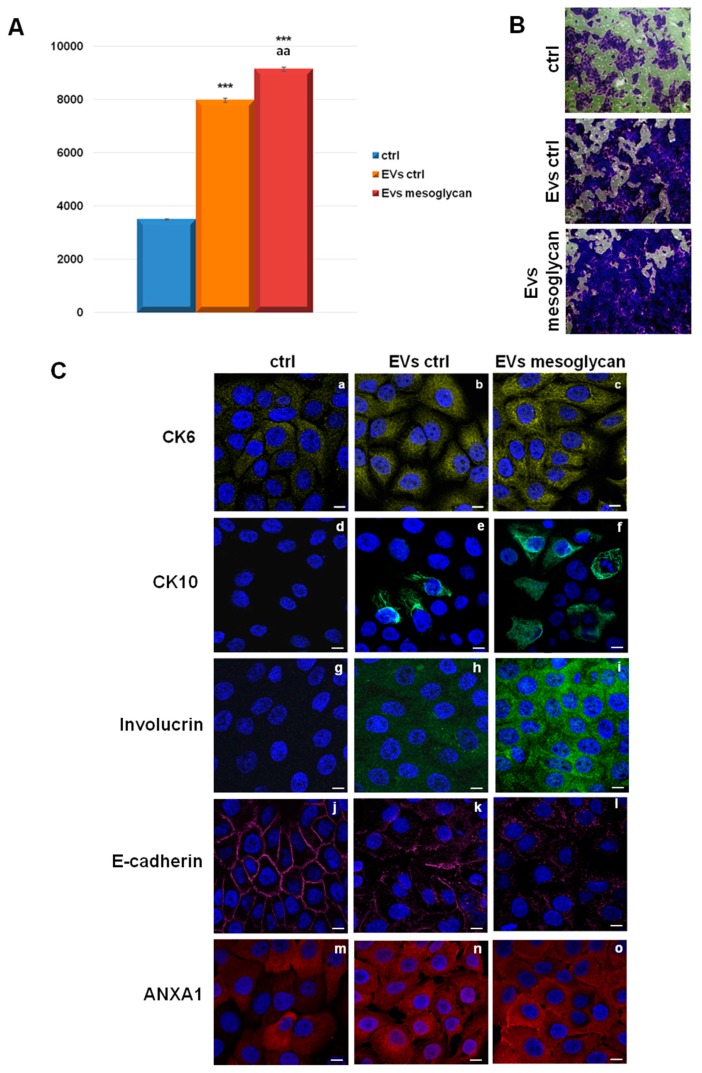
(**A**) Invasion assay on keratinocytes treated with control EVs and EVs released by keratinocytes treated with mesoglycan 0.3 mg/mL. *** *p* < 0.001 for EVs ctrl and EVs mesoglycan vs. non treated control; aa *p* < 0.01 for EVs mesoglycan vs. EVs ctrl. This mean derived from cell counts of 10 separate fields per well ± SEM of three experiments with similar results. (**B**) Bright field representative images of invasion assay. (**C**) Immunofluorescence analysis to detect CK6 (panels a–c), CK10 (panels d–f), involucrin (panels g–i), E-cadherin (panels j–l) and ANXA1 (panels m–o) on keratinocytes treated or not with EVs ctrl and EVs mesoglycan. Magnification 63 × 1.4 NA (numerical aperture). Bar = 20 μm. The data are representative of three experiments with similar results.

As reported in Figure 2A, both types of EVs were able to increase the levels of keratinocyte invasion, compared to untreated cells. Furthermore, a significant increase of invasion behaviour of HaCaT treated with EVs mesoglycan compared with EVs ctrl was observed. 

During wound healing, the differentiation of the keratinocytes occurs as one of the final steps [8].

Therefore, EVs ctrl and EVs mesoglycan were administered to keratinocytes in order to observe their effects on some proteins involved in this process. Immunofluorescences in Figure 2B show that EVs ctrl and EVs mesoglycan induced a significant increase of cytokeratins 6 and 10 (CK6 and CK10) and involucrin. In parallel, we found the loss of the typical membrane localization by E-cadherin and its slight decreased expression in presence of the microvesicles. Our confocal analysis further showed that EVs mesoglycan preserved a stronger effect in the promotion of keratinocytes differentiation compared to EVs ctrl. 

Finally, ANXA1 increased its expression and translocated to the plasma membrane of HaCaT cells treated with EVs mesoglycan more than EVs ctrl. In Figure S1 the quantitative analysis of protein levels through immunofluorescence assays is shown.

### 3.3. The ANXA1 Mimetic Peptide Induced the Translocation of ANXA1 to the Keratinocytes Plasma Membrane and Its Externalization

**Figure 3:**
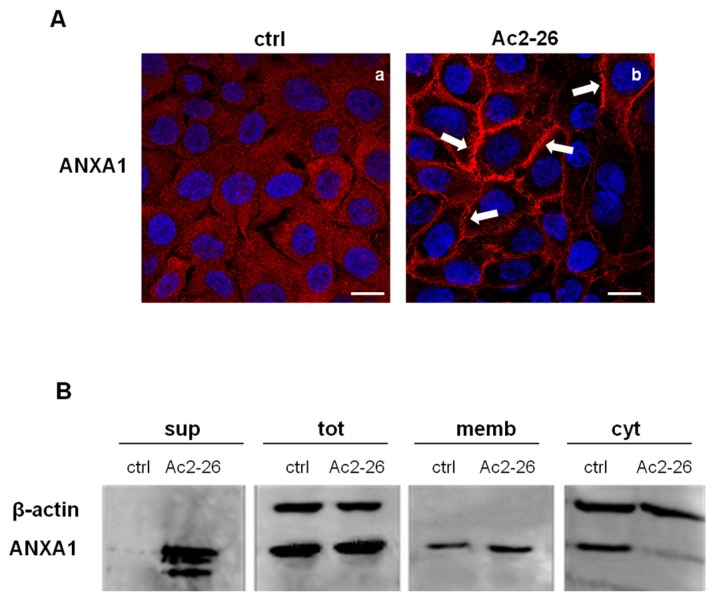
(**A**) Immunofluorescence analysis to detect ANXA1 in keratinocytes not treated (a) and treated with Ac2-26 1 μM (panel b). Magnification 63 × 1.4 NA. Bar = 20 μm. (**B**) Extracellular, whole, membrane, and cytosol expression of ANXA1 in HaCaT cells treated or not with Ac2-26 1 μM was analysed by Western blot with an anti-ANXA1 antibody. Protein extracts from cellular compartments were obtained as described in Materials and Methods Section. Protein normalization and the check of the sample quality were performed on β-actin levels. The data shown in (**A**) and (**B**) are representative of three experiments with similar results.

Based on the results shown in Figure 1C, we focused on the possible role of extracellular ANXA1 on keratinocytes. We used the ANXA1 N-terminal peptide, Ac2-26, to mimic its biological activities, as previously reported [20]. Thus, immunofluorescence analysis in Figure 3A shows that ANXA1 moved to the plasma membrane in HaCaT cells treated with Ac2-26 (panel b, white arrows), by compared there was a diffuse signal in not treated cells (panel a). This result has been confirmed by Western blot (Figure 3B). Particularly, we found the ANXA1 signal at different molecular weights in the supernatant of cells treated with Ac2-26. In the same experimental point, we show the increased expression of ANXA1 at the plasma membrane and the related reduction in the cytosol. Compartimentalized protein extraction has been performed as reported in Material and Methods section.

### 3.4. Ac2-26 Peptide Promoted Keratinocytes Motility through FPRs 

The localization of ANXA1 on the cell surface and its externalization are important events encouraging cell motility [23]. Furthermore, several evidences report that the main functions of the secreted ANXA1 are triggered through the activation of FPRs [22,23,26,27,28,29]. For these reasons, we performed the Wound Healing and invasion assays on keratinocytes in the presence of Ac2-26 with or without Boc-1, a pan-antagonist of FPRs (namely able to block FPR-1, FPR-2, FPR-3) at a concentration of 100 µM [30].

**Figure 4:**
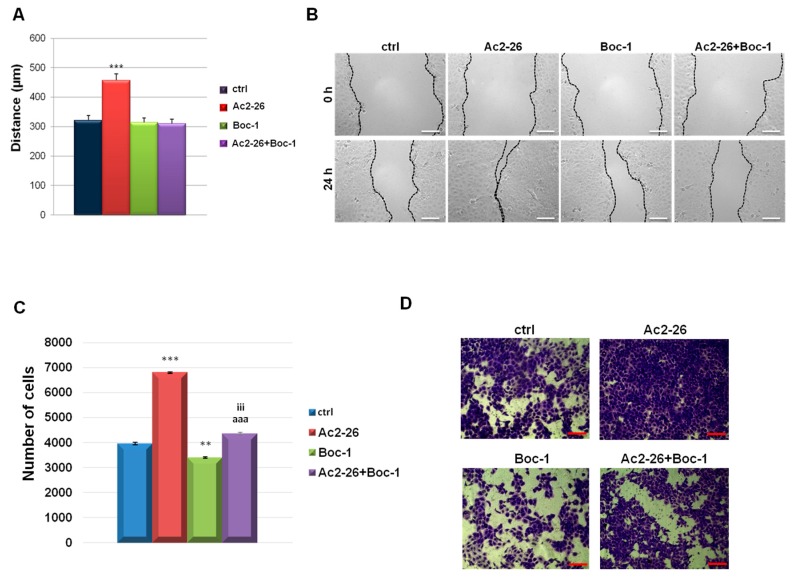
(**A**) Analysis of the migration rate in the presence or not of Ac2-26 (1 µM) and Boc-1 (100 µM). The migration rate was determined by measuring the wound closure by individual cells from the initial time (0 h) to the selected time-points (24 h) (bar of distance tool, Leica ASF software). Representative images for migration assay are reported in (**B**). Bar = 150 µm. (**C**) Analysis of invasion speed of keratinocytes treated or not with Ac2-26 (1 µM) and Boc-1 (100 µM). Data represent the mean cell counts of 10 separate fields per well. The data represent a mean of three independent experiments ± SEM, their statistical significance were evaluated using Student’s *t*-test, assuming a 2-tailed distribution and unequal variance. ** *p* < 0.01; *** *p* < 0.001 for treated vs. non treated cells; aaa *p* < 0.001 for Ac2-26+Boc-1 vs. Ac2-26; iii *p* < 0.001 for Ac2-26+Boc-1 vs. Boc-1. (**D**) Representative images of analysed fields of invasion assay on keratinocytes. Magnification 20×. Bar = 50 μm.

Results in Figure 4A show a progressive increase in migration speed of cells treated with the ANXA1 mimetic peptide, compared to control ones. The stimulation of cell migration by Ac2-26 was inhibited by Boc-1. Similarly, the invasive ability of the keratinocytes is positively influenced by Ac2-26, but in the presence of Boc-1 the number of the cells invading the coating of matrigel, is strongly reduced (Figure 4C). Both for migration and for invasion processes, the results are further reported in Figure 4B,D, respectively, representing bright field pictures.

### 3.5. Ac2-26 Induced Early Differentiation in Keratinocytes through FPRs

**Figure 5:**
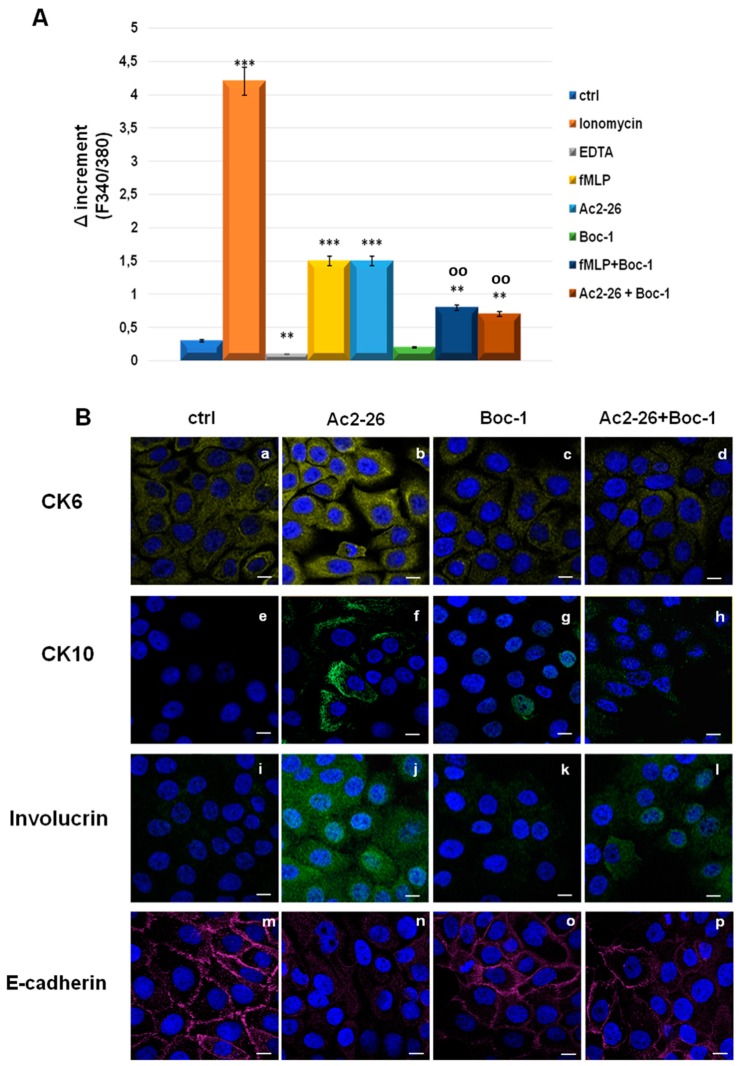
Effects of ionomycin (1 mM), EDTA (15 mM), fMLP (50 nM), Ac-2-26 (1 μM) and Boc-1 (100 μM) on the Formyl Peptide Receptor (FPR)-induced rise in intracellular Ca^2+^ in HaCaT cells. (**A**) The histograms show the fluorescence ratio calculated as F340/F380 nm in absence of extracellular Ca^2+^. Data are means ± SEM of three experiments with similar results. ** *p* < 0.01; *** *p* < 0.001 treated cells vs. non treated control; oo *p* < 0.01 fMLP + Boc-1 and Ac2-26 + Boc-1 vs. fMLP and Ac2-26, respectively. (**B**) Immunofluorescence analysis to detect: CK6 (panels a–d), CK10 (panels e–h), involucrin (panels i–l), E-cadherin (panels m–p) treated or not with Ac2-26 with or without Boc-1. Magnification 63 × 1.4 NA. Bar = 20 μm.

It is known that the interaction between ANXA1 and FPRs causes a series of cellular responses, such as the ERK phosphorylation and the increase in intracellular [Ca^2+^] concentration [20]. Calcium levels in human keratinocytes are important for the differentiation processes [31]. Therefore, we examined stimulated release of calcium from intracellular reserves. The keratinocytes were incubated with the fluorescent calcium indicator FURA-2 AM before stimulation with ionomicyn (1 mM) as positive control, EDTA (15 mM) as a negative one, fMLP the natural FPR agonist (50 nM), Ac2-26 (1 μM), Boc-1 (100 μM) and fMLP/Boc-1 and Ac2-26/Boc-1 together. The histogram in Figure 5A shows that fMLP and Ac2-26 are able to increase intracellular calcium levels. Calcium mobilization was not observed in cells treated with the two peptides together with the FPR antagonist Boc-1.

Next, performing confocal analysis on the typical markers of the early differentiation, we show that the administration of Ac2-26 induced the increase of the expression of CK6 (panels b), CK10 (panel f), and involucrin (panel j) (Figure 5B). At the same time, the localization of E-cadherin disappeared from the membrane when the keratinocytes were treated with Ac2-26 (Figure 5B, panel n). Furthermore, there were no changes in protein localization and/or expression in presence of Boc-1 (panels c, g, k, o). Finally, it was interesting to observe that the effects induced by the ANXA1 mimetic peptide were partially reverted by Boc-1 inducing an intermediate phenotype between what has been shown by Ac2-26 and Boc-1 alone (panels d, h, l, p). The quantification of fluorescence intensity of the protein shown in Figure 5B is reported in Figure S2.

### 3.6. EVs-Containing ANXA1 Improved the Invasive Behaviour of Keratinocytes through FPRs

**Figure 6:**
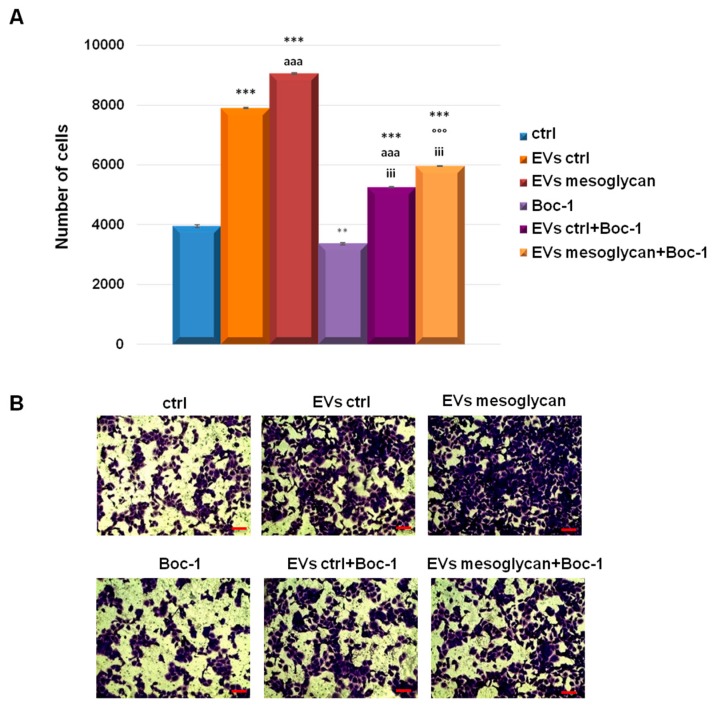
(**A**) Analysis of invasion speed of keratinocytes treated or not with EVs ctrl, EVs mesoglycan and Boc-1 (100 µM). Data represent the mean cell counts of 10 separate fields per well ± SEM of 3 experiments with similar results. ** *p* < 0.01; *** *p* < 0.001 treated cells vs. non treated control; aaa *p* < 0.001 EVs ctrl + Boc-1 vs. EVs ctrl; °°° *p* < 0.001 EVs mesoglycan + Boc-1 vs. EVs mesoglycan and iii *p* < 0.001 EVs ctrl + Boc-1 and EVs mesoglycan + Boc-1 vs. Boc-1. (**B**) Representative images of analysed fields of invasion assay on keratinocytes. Magnification 20×. Bar = 50 μm.

Based on the results obtained to date in this study about the roles of EVs and Ac2-26, an invasion assay using EVs and Boc-1 together was performed. First, we confirmed the autocrine pro-invasive activity of EVs mesoglycan more than EVs ctrl on HaCaT cells. Then, we found a significant reduction of keratinocyte invasive speed in presence of the FPRs pan-antagonist Boc-1 even when this compound was administrated together with the two kinds of EVs. These results were represented with the histogram in Figure 6A and the related bright filed images in Figure 6B.

### 3.7. ANXA1 Contained in EVs Induced the Differentiation of Keratinocytes through FPRs

As shown for the invasive ability of HaCaT cells, the autocrine effect of ANXA1 as a component of EVs has been studied also for the differentiation process. Initially, we assessed the release of calcium from intracellular reserves. By the fluorescent probe FURA-2 AM we found a significant increase in calcium levels in cytosol in the presence of EVs ctrl and much more of EVs mesoglycan. We also assisted a notable rescue when both EVs have been administrated together with Boc-1. We used ionomycin and EDTA as technical controls.

**Figure 7:**
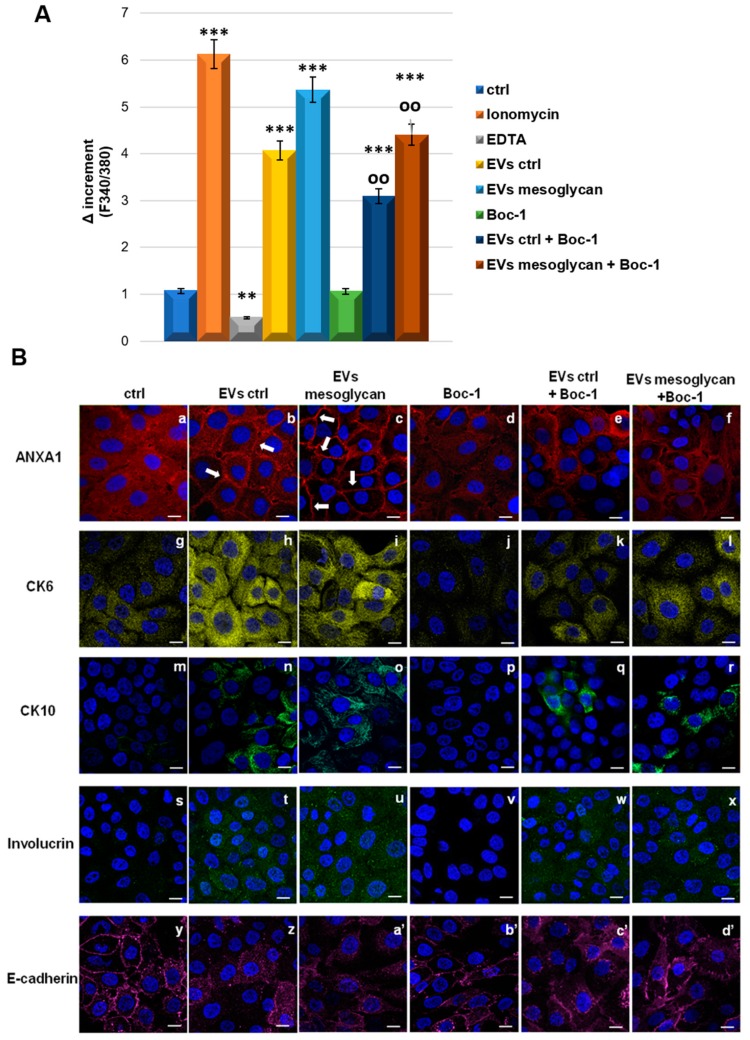
(**A**) Effects of ionomicyn (1 mM), EDTA (15 mM), EVs ctrl (20 μg). EVs mesoglycan (20 μg) Boc-1 (100 μM), EVs ctrl+Boc-1 and EVs mesoglycan+Boc-1 on the variation of intracellular Ca^2+^ levels in HaCaT cells. The histograms show the fluorescence ratio calculated as F340/F380 nm in absence of extracellular Ca^2+^. Data are means ± SEM of three experiments with similar results. ** *p* < 0.01; *** *p* < 0.001 treated cells vs. non treated control; oo *p* < 0.01 EVs ctrl + Boc-1 and EVs mesoglycan + Boc-1 vs. EVs ctrl and EVs mesoglycan, respectively. (**B**) Immunofluorescence analysis to detect ANXA1 (panels a–f), CK6 (panels g–l), CK10 (panels m-r), involucrin (panels s–x) and E-cadherin (panels y-d’) on keratinocytes treated or not with EVs ctrl, EVs mesoglycan and Boc-1. Magnification 63 × 1.4 NA. Bar = 20 μm. The data are representative of three experiments with similar results.

Additionally, in presence of EVs ctrl and EVs mesoglycan, we examined the increased expression of ANXA1 accompanied by the translocation to cell surface (Figure 7 panels a–c), the increment of levels of CK6 (panels g–i), CK10 (panels m–o) and involucrin (panels s–u), finally, the disorganization of E-cadherin far from the plasma membrane (panels y–a’). All these events did not occur in the presence of Boc-1 (panels d, j, p, v, b’). Interestingly, the positive effect on the differentiation was partially reverted when both kinds of microvesicles were administered to the keratinocytes together with Boc-1 (panels e, f for ANXA1, panels k, l for CK6, panels q, r for CK10, panels w, × for involucrin and panels c, d’ for E-cadherin). Indeed in this case we found an intermediate phenotype between cells treated with EVs and Boc-1 alone. In Figure S3 the quantitative analysis of protein levels through immunofluorescence assays is shown.

## 4. Discussion

A new molecular mechanism of skin wound repair by mesoglycan fibrinolytic drug has been previously observed in our laboratory. Indeed, this mixture of GAGs has been studied in vitro and has shown significant effects favouring keratinocytes, fibroblasts, and endothelial cells activation [5,6]. Interestingly, the effects of mesoglycan on HaCaT cells were mediated by the co-receptor SDC4 which triggers the phosphorylation of protein kinase Cα (PKCα), inducing migration, invasion, and early differentiation [8]. HaCaT cells, as normal human immortalized keratinocytes, represent a good in vitro model to study the differentiation process since they can revert back between a differentiated and a basal state depending on specific stimuli.

On the other hand, the considerable burden on the healthcare system of wound healing has suggested a new kind of therapy based on vesicles and exosomes. This experimental treatment could overcome the limitation of cellular therapy [32]. Over the past few years, it has been found that the local injection of exosomes, also through an hydrogel formulation, into skin wounds in mice resulted in accelerated re-epithelialization, reduced scar widths, and enhanced new blood vessel formation [16,25,33,34]. Particularly, a study highlighted that EVs isolated from HaCaT cells stimulate cell migration in an autocrine manner thanks to their protein component [35].

Based on this emerging role of extracellular microvesicles and on the knowledge that SDC4 is involved in the exosome biogenesis [17], we investigated a further in vitro mechanism downstream of the action of mesoglycan. Thus, we found that this compound allows the secretion of extracellular vesicles, enriched in exosomes, by keratinocytes. HaCaT cells are able to produce exosomes but here we highlighted that in presence of mesoglycan this process is increased. Moreover, these EVs significantly intensify keratinocyte cell invasion speed and differentiation suggesting a strong autocrine effect. The two processes we have analysed occur at about 8–24 h after skin injury, when keratinocytes migrate to the wounded area and undergo a rapid proliferation phase that covers the wound by forming a cell monolayer. Finally, a cell cycle arrest and the launch of the differentiation process take place [1]. Generally, the microvesicles, as exosomes, can exert these phenomena thanks to their content of proteins, nucleic acids, and lipids belonging to the parent cell [36].

Our previous work has shown that mesoglycan induced the translocation to the inner surface of the plasma membrane of ANXA1 followed by its secretion in extracellular environments [8]. Furthermore, since ANXA1 is one of the proteins responsible of exosome formation and secretion [15], we have hypothesised that ANXA1 could be secreted by exosomes. This process is confirmed in this study in our in vitro system. Moreover, a great number of works report that the extracellular form of ANXA1 is able to act in an autocrine or paracrine/juxtacrine manner through its interaction with FPRs triggering several biological effects as cell motility both in physiological and pathological systems [22,23,26,27,28,29].

The pro-resolving effect of ANXA1 in wound healing has been deeply reported, particularly through FPRs [14,37,38]. Leoni and colleagues showed that the synthetic vesicles containing Ac2-26 were able to increase the closure of mice intestinal mucosa lesions miming the effects of the complex ANXA1/EVs [39]. Additionally, it has been reported that FPRs are involved in keratinocytes activation [40].

In this study, we show for the first time that the ANXA1 mimetic peptide Ac2-26 triggers a positive loop enhancing the translocation of ANXA1 to the plasma membrane and its following externalization. Furthermore, the stimulation of keratinocyte motility and differentiation is due to the activation of FPR pathway. In this way, we could show that the ANXA1-containing EVs induced the analysed processes acting on FPRs. 

Interestingly, in most processes involving extracellular ANXA1, other ligands presumably mediate cell surface binding and localization. Among these molecules, GAGs appear as known ligands to mediate ANXA1 attachment to cell surfaces [41,42]. Notably, the interaction between GAGs and other annexins has been also demonstrated through cocrystal structures [43,44]. Thus, we can speculate that mesoglycan could form a complex with ANXA1 further facilitating the convergence of this protein to its receptor partners on the outer cell surface and enhancing the triggered effects.

**Figure 8:**
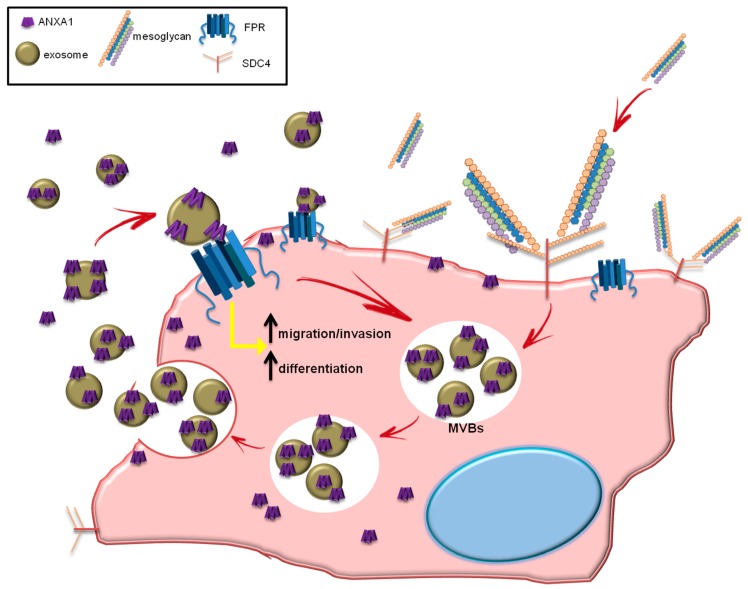
The mesoglycan administered to keratinocytes, interacts with the co-receptors SDC4 through its heparan sulfate components by activating a series of signal transduction events leading to cellular activation. This binding activates SDC4 and promotes the formation of multivesicular bodies (MVBs) which are transported to the plasma membrane for exosome release. The microvesicles released are enriched of all the content of the parent cell, including ANXA1 protein. ANXA1 interacts with FPRs to promote keratinocyte migration and invasion and differentiation. The internal loop triggered by ANXA1 leads to the formation and the release of EVs, amplifying the biological process.

Taken together, our data suggest that the axis ANXA1/EVs/FPRs represents an autocrine loop able to promote the activation of keratinocytes, a fundamental event of the re-epithelialization during wound repair. Additionally, the obtained results allowed us to speculated about a new mechanism of action by which EVs amplify the beneficial effects of mesoglycan. In this loop ANXA1 represents the link for the activity of this mixture of GAGs. In Figure 8 the circuit we suggest is summarized.

In conclusion, in this work we have focused on the autocrine effects of the complex ANXA1/EVs/FPRs induced by mesoglycan. Here, we found a new important tile in the explication of the mechanism of action of this compound previously used exclusively as a fibrinolytic drug. Nevertheless, an interesting issue would be the investigation of the paracrine function on other cell populations acting in wound repair as fibroblasts, endothelial cells or granulocytes. Further studies are needed to describe this topic and to translate these finding in an in vivo system in order to better define the biochemical mechanism at the base of the activation of keratinocytes and of other cell populations recruited in tissue damage repair processes.

## Supplementary Materials

The following are available online at https://www.mdpi.com/2073-4409/8/7/753/s1, Figure S1: Quantification of immunofluorescence shown in Figure 2 (A) CK6 ((a)–(c)); (B) CK10 ((d)–(f)); (C) involucrin ((g)–(i)); (D) E-cadherin ((j)–(l)); (E) ANXA1 ((m)–(o)) as percent of fluorescence intensity per cell area. * *p* < 0.05, ** *p* < 0.01, *** *p* < 0.001 treated vs. not treated cells, Figure S2. Quantification of immunofluorescence shown in Figure 5 (A) CK6 ((a)–(d)); (B) CK10 ((e)–(h)); (C) involucrin ((i)–(l)); (D) E-cadherin ((m)–(p)) as percent of fluorescence intensity per cell area. *** *p* < 0.001 treated vs. not treated cells, Figure S3. Quantification of immunofluorescence shown in Figure 7 (A) ANXA1 ((a)–(f)); (B) CK6 ((g)–(l)); (C) CK10 ((m)–(r)); (D) involucrin ((s)–(x)); (E) E-cadherin ((y)–(d’)) as percent of fluorescence intensity per cell area. * *p* < 0.05, ** *p* < 0.01, *** *p* < 0.001 treated vs. not treated cells.
