# Supplementary figures and images for: Annexin A1 Contained in Extracellular Vesicles Promotes the Activation of Keratinocytes by Mesoglycan Effects: An Autocrine Loop Through FPRs
Source: Cells. 2019 Jul 19;8(7):753. doi: 10.3390/cells8070753 (PMC6679056; doi:10.3390/cells8070753)

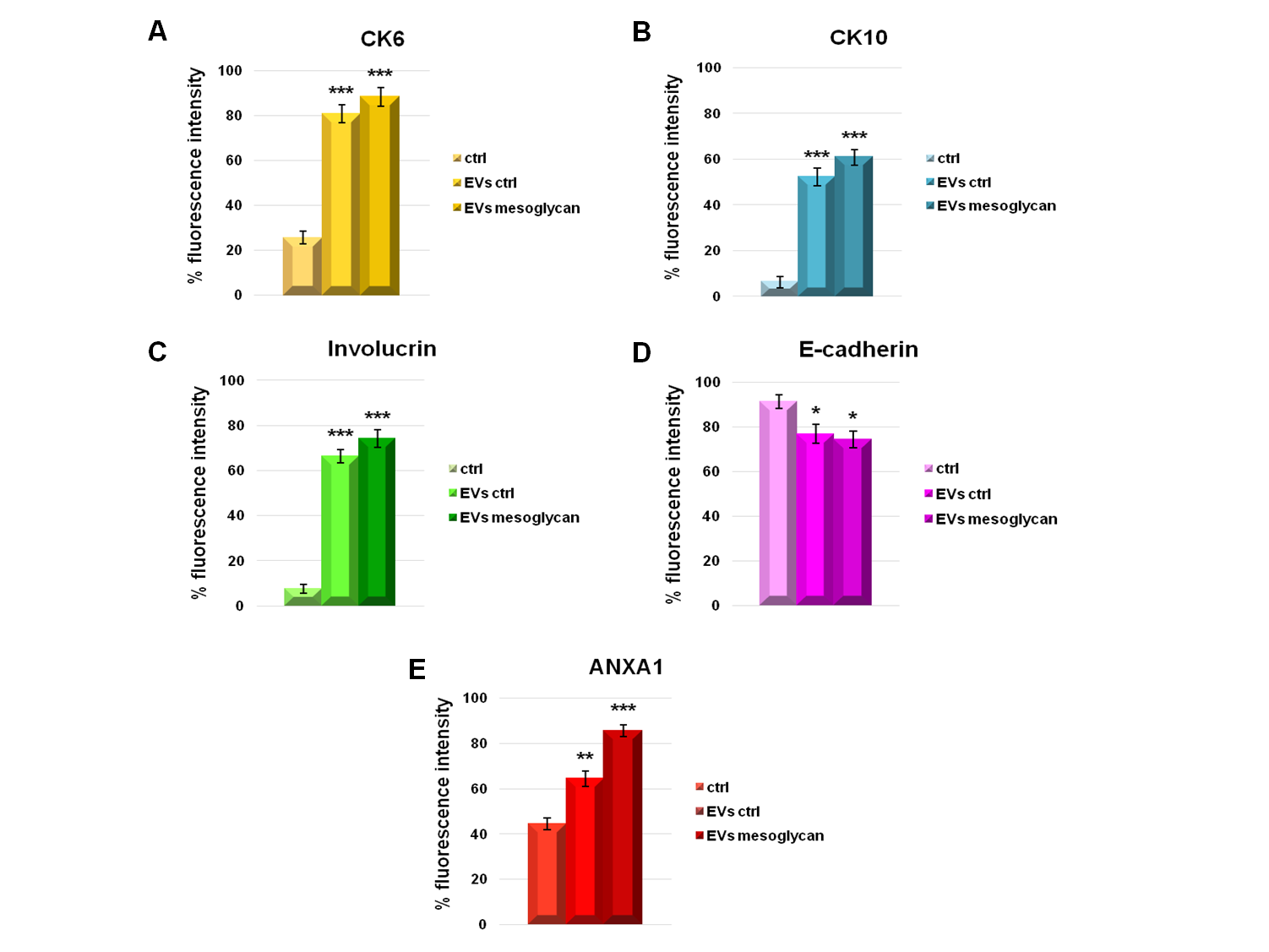

Supplement: Supplementary file 1 [file cells-08-00753-s001.zip › figure S1.tif]

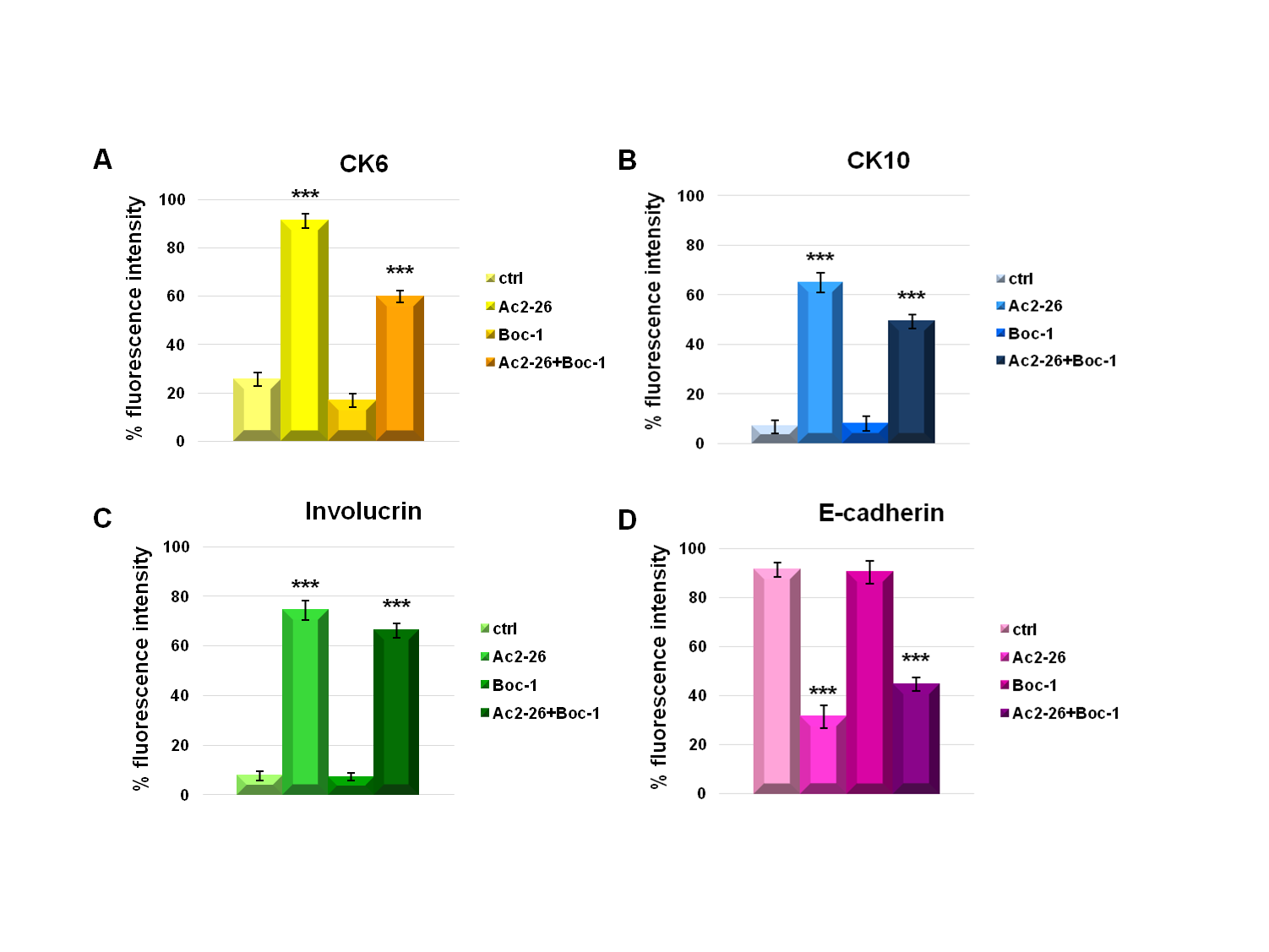

Supplement: Supplementary file 1 [file cells-08-00753-s001.zip › figure S2.tif]

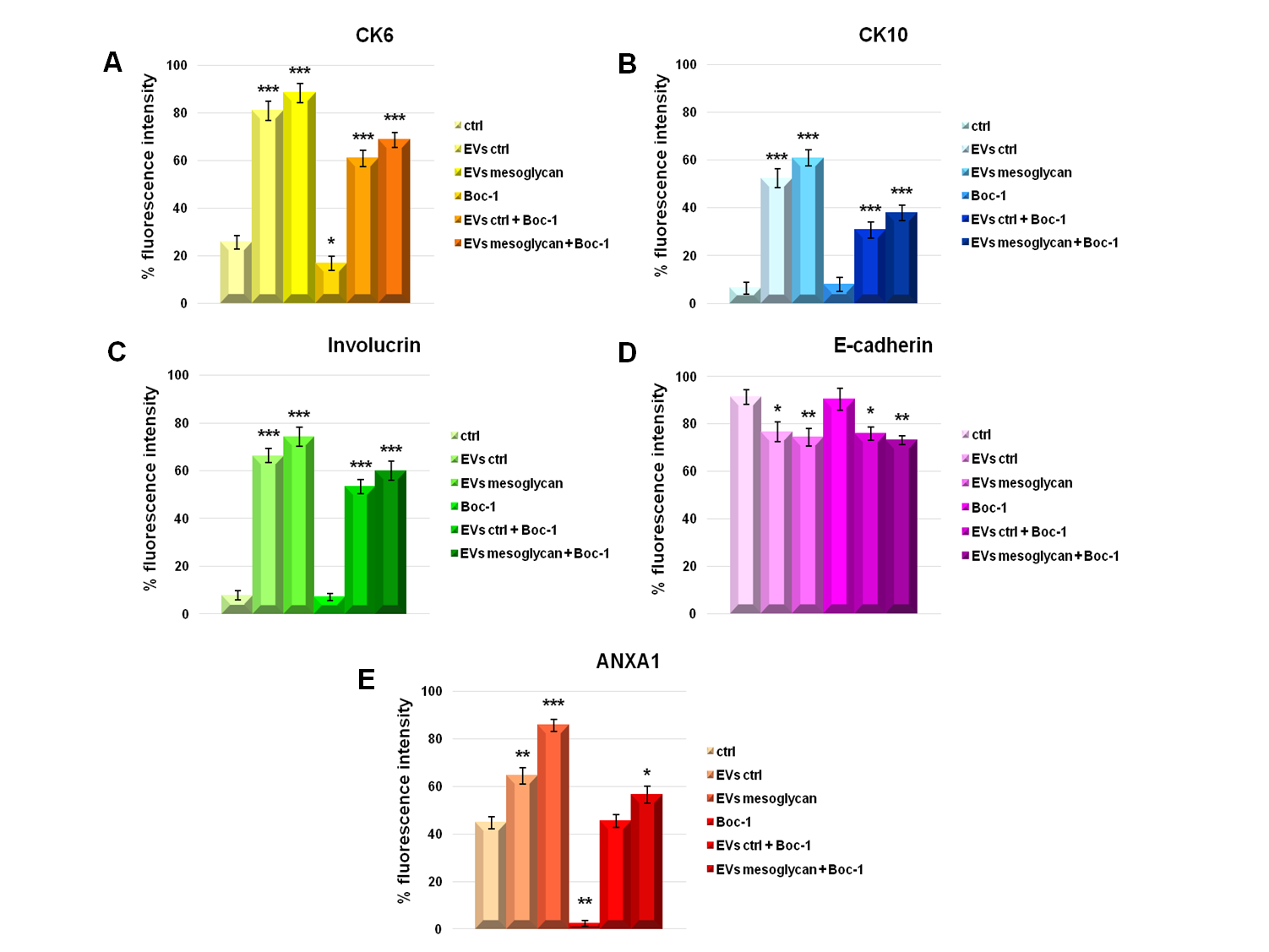

Supplement: Supplementary file 1 [file cells-08-00753-s001.zip › figure S3.tif]
